# Tiny Yet Indispensable Plant MicroRNAs Are Worth to Explore as Key Components for Combating Genotoxic Stresses

**DOI:** 10.3389/fpls.2019.01197

**Published:** 2019-10-04

**Authors:** Moumita Roy Chowdhury, Jolly Basak

**Affiliations:** ^1^Computational Structural Biology Lab, Department of Biotechnology, Indian Institute of Technology Kharagpur, Kharagpur, India; ^2^Laboratory of Plant Stress Biology, Department of Biotechnology, Visva-Bharati, University Santiniketan, India

**Keywords:** UV radiation, genotoxic stress, microRNAs, DNA lesions, DNA damage response network

## Abstract

Plants being sessile are always exposed to various stresses including biotic and abiotic stresses. Some of these stresses are genotoxic to cells causing DNA damage by forming lesions which include altered bases, cross-links, and breaking of DNA strands, which in turn hamper the genomic integrity. In order to survive through all these adverse conditions, plants have evolved different DNA repair mechanisms. As seen from the mammalian system and different human diseases, various microRNAs (miRNAs) can target the 3′-untranslated region of mRNAs that code for the proteins involved in DNA repair pathways. Since miRNAs play an important role in plant cells by regulating various metabolic pathways, it can also be possible that miRNAs play an important role in DNA repair pathways too. However, till date, only a handful of plant miRNAs have been identified to play important role in combating genotoxic stresses in plants. Limitation of information regarding involvement of miRNAs in DNA repair as well as in ROS scavenging prompted us to gather information about plant miRNAs specific for these tasks. This mini-review aims to present pertinent literature dealing with different genotoxic stresses that cause genome instability as well as plant specific responses to survive the damage. This is intertwined with the involvement of miRNAs in genotoxic stress in plants, challenges of applying miRNAs as a tool to combat DNA damage along with ways to overcome these challenges, and finally, the future prospective of these understudied aspects.

## Introduction

Plants are always subjected to various environmental stresses which cause severe DNA damage along with genotoxic stress, which in turn may reduce the development, genome stability, and crop productivity. Drought, extreme temperature stress, salt stress, oxidative stress, and damage due to UV irradiation are the abiotic stresses encountered by the plants on daily basis (Tuteja et al., 2011). Plants are also exposed to several biotic stresses through infection by bacteria, virus, pathogens, fungi, and insects (Huang et al., 2016). These genotoxic stresses cause serious damages to plant genome and put the genome integrity at risk (Tuteja et al., 2009).

There are numerous DNA-damaging agents including but not limited to bromouracil, nitrous acid, ethyl methane sulfonate, ethidium bromide like chemical mutagens, or ROS molecules, such as hydrogen peroxide (H_2_O_2_), superoxide [$\left({\text{O}}_{2}^{-}\right)$], hydroxyl radical (^.^OH), and even different types of radiations (UV rays, gamma rays, X-rays) (Tuteja et al., 2001). Chemical mutagens damage DNA by altering DNA structure, base pairing, and base structure along with frameshift mutation. UV radiation from sunlight, consisting of UV-A, UV-B, and UV-C types of radiation, causes DNA damage by producing pyrimidine photodimers including cyclobutane pyrimidine dimers (CPD) (Hu and Adar, 2017). These pyrimidine dimers inhibit transcription and replication and induce oxidative stress. ROS overproduction is toxic to plant cells causing damage to DNA, lipids, cell membranes, and proteins (Caverzan et al., 2019).

Plants have evolved strategies to withstand continuous DNA damage and to maintain genome stability. Photoreactivation is the major DNA repair pathway in which the lesions induced by UV radiation are directly reversed back to its normal form (Friedberg, 2015). Single-strand breaks, deaminated, oxidized, or alkylated bases, are repaired by base excision repair (BER) while nucleotide excision repair (NER) works to repair CPDs, although with low capacity (Ries et al., 2000). Homologous recombination (HR) and nonhomologous end-joining (NHEJ)—mediated pathways help in repairing double-strand breaks (Spampinato, 2017). Plants have also evolved mechanisms to scavenge oxidative stress-generated free radicals through different enzymatic processes involving catalases (CATs), peroxidases (POXs), and superoxide dismutases (SODs), as well as non-enzymatic processes involving ascorbic acid and secondary metabolites (Das and Roychoudhury, 2014).

microRNAs (miRNAs) are 20–24 nucleotides long, small non-coding ribonucleic acids, involved in the regulation of gene expression by interfering with various post-transcriptional processes (Yu et al., 2017). Plant miRNAs play vital roles in growth and development as well as in tolerating several types of biotic and abiotic stresses like extreme temperatures, nutrient deprivation, and salinity (Li et al., 2016). Plant miRNAs control gene expression either by cleavage of the target mRNA or through translational inhibition (Xie et al., 2015). miRNAs specifically identify targets by base complementarity and, in turn cleave, translationally repress or destabilize the target mRNAs (Moro et al., 2018). Perfect base pairing of miRNA with target mRNA leads to the cleavage of the targets, whereas the imperfect binding results in translational repression of the target mRNAs (Djami-Tchatchou et al., 2017).

A highly controlled regulation is required to maintain the DNA damage response (DDR) network and ROS scavenging mechanisms to combat genotoxic stresses in plant cells. It is yet to explore whether plant miRNAs play substantial roles in regulating the expression of the genes that are directly or indirectly involved in genotoxic stresses. The fact that only a handful of studies have considered the involvement of miRNAs in DDR and ROS scavenging prompted us to gather information about plant miRNAs specific for these functions. However, we have faced several hurdles in this task due to the unavailability of miRNAs and direct involvement of their corresponding targets within the DDR network. Based on the available information, we have discussed about different genotoxic stresses in plants, role of plant miRNAs in combating genotoxic stresses, hurdles in applying miRNAs as a tool to combat genotoxic stresses in plants, and ways to overcome these problems. The gathered information will be helpful for future practical application of miRNAs as potential tools to secure and stabilize crop yield in view of the continuous climatic changes.

## Genotoxic Stresses Leading to Instability in Plant Genome

There are numerous DNA-damaging processes continuously threatening the integrity of the plant genome, including various chemical mutagens and UV radiation, the latest being amongst the most hazardous. These types of stresses generate various DNA lesions that includes altered, missing, and mismatched bases; single- or double-strand breaks; insertion or deletion of bases; pyrimidine dimers; and cross-linked DNA strands, which are genotoxic to plant cells (Tuteja et al., 2001). These damages in turn inhibit transcriptional and translational processes which ultimately affect plant growth and crop yield.

### DNA Damage Due to the Production of Free Radicals

Plant cells get damaged by the excess production of ROS which includes free radicals like H_2_O_2_, [${\text{O}}_{2}^{-}$], and.OH (Inzé and Montagu, 1995; Sharma et al., 2012). Although [${\text{O}}_{2}^{-}$] and H_2_O_2_ can damage the DNA, these two radicals are very unstable and can easily be removed from the system in the absence of metal catalysts (Tuteja et al., 2009). Conversion of [${\text{O}}_{2}^{-}$] and H_2_O_2_ to.OH is catalyzed by metals, and.OH is the major source of toxicity in the cells as it reacts with almost all the cellular macromolecules including DNA (Sharma et al., 2012). Lipid peroxidation can also induce the production of ROS which leads to cross-linking of DNA and proteins; hence, they are toxic and mutagenic for cells (Gaschler and Stockwell, 2017). The reactive electrophiles are responsible for the production of various DNA adducts, namely, propano adducts, adducts of acrolein, and crotonaldehyde. The 4-hydroxynonenal compound is the most genotoxic whereas malondialdehyde (MDA) is considered the most mutagenic products of lipid peroxidation (Łuczaj and Skrzydlewska, 2003).

### DNA Damage Induced by UV Radiation

UV radiation plays an important role in damaging plant genome stability by producing pyrimidine hydrates as a result of oxidative damage and cross-links between DNA and/or protein, and in turn, inhibits plant growth and development (Gill et al., 2015). UV-B, being the most harmful form of UV radiation, is responsible for the production of DNA lesions like CPD and pyrimidine (6–4) pyrimidinone adducts (6–4 PPs) (Ries et al., 2000; Law et al., 2013). CPDs are found to block transcribing complexes, which in turn is responsible for the alteration of gene expression patterns. In addition to CPD-mediated damage, UV-B also induces delay of G1-to-S phase transition within the plant cell cycle (Jiang et al., 2011). UV-C induces both single-stranded breaks and double-stranded breaks in *Arabidopsis* (Abas et al., 2007). Oxidative DNA damages are also found to be responsible for UV-associated mutagenicity and instability of plant genome (Manova and Gruszka, 2015).

## Plant Cellular Responses to DNA Damage

Plants respond to DNA damage by activating a complex DDR network consisting of mechanisms like DNA repair, cell cycle arrest, and apoptosis (Yoshiyama et al., 2013). Different types of DNA repair mechanisms like photoreactivation, BER, NER, mismatch repair (MMR), and double-strand break (DSB) repair gets activated in plants in response to DNA damage (Kimura and Sakaguchi, 2006). It is found that large numbers of protein components are involved in these repair mechanisms, a handful of which being potential target of miRNAs. The involvements of some of these protein components in the repair mechanisms are briefly discussed.

In the photoreactivation-mediated DNA repair mechanism, thymine dimer structures are found to be cleaved by CPD lyase or (6–4) photolyase (Waterworth et al., 2002). Photolyases bind specifically to the damaged site of double-stranded DNA in a light independent manner, although it gets activated through UV-A for correction of the lesions. This is followed by the splitting of the covalent bonds of the dimers in an error-free manner (Manova and Gruszka, 2015).

Within BER, apurinic/apyrimidinic sites are found to be recognized by lesion-specific DNA glycosylases which cleave the N-glycosidic bond following the removal of the affected base and the generation of abasic sites in plants (Manova and Gruszka, 2015). In *Arabidopsis*, carrot, and rice, several DNA glycosylases were identified—as a couple of examples, 3-methyladenine-DNA glycosylase (MAG), formamido-pyrimidine-DNA glycosylase (FPG) 8-oxoG DNA-glycosylases (OGG), uracil-DNA glycosylase (UNG), and DNA glycosylase/lyase DNG701 (Santerre and Britt, 1994; Dany and Tissier, 2001; Talpaert-Borlé and Liuzzi, 2005; La et al., 2011). NER recognizes and repairs several types of DNA lesions induced by UV-rays and other mutagens, and the process was extensively studied in *Arabidopsis thaliana*. *Xeroderma pigmentosum* complementation of group C (XP-C)/AtRAD4 recognizes DNA damage. This is followed by the unwinding of DNA containing damaged portion by the transcription factor IIH (TFIIH), including AtXP-D. Damaged oligonucleotides get excised by AtXP-F or other *Arabidopsis* homologs (e.g., AtERCC1). The excised gap is filled through the activity of proliferating cell nuclear antigen (PCNA) and replication factor C (RFC)–mediated DNA synthesis. Finally, DNA ligase I joins the DNA strands (Xu et al., 1998; Ishibashi et al., 2003; Kimura and Sakaguchi, 2006; Molinier et al., 2008; Liu et al., 2012).

Within MMR, homologs of MutS (MSH) recognize the mismatch generated by the incorporation of incorrect bases by DNA polymerase. This is followed by generation of nicks through the activity of MutL (MLH) homologues and the successive steps of the repair system (Dion et al., 2007; Lario et al., 2015).

DSBs are repaired in plants by HR- and NHEJ-mediated pathways (Puchta, 2005). In *Arabidopsis* and rice, many components of DSB repair mechanisms have been identified—for example, AtRad51, AtRadA, AtRad50, OsRadA, AtMre11, AtKu70, AtKu80, *Arabidopsis* DNA ligase IV, AtXRCC4, AtXP-F, and AtERCC1 (Manova and Gruszka, 2015). Additionally, it has been discovered that Ataxia telangiectasia–mutated (ATM) and ATM-Rad3-related (ATR) proteins play important roles in DNA repair. Checkpoint kinases (CHK), including Chk1 and Chk2, work downstream of ATM and ATR proteins, where activated ATR initiates G-2 phase arrest by phosphorylating CHK1. This in turn is responsible for DDR-induced transcriptional repression (Culligan et al., 2004).

## Involvement of Plant MiRNAs in Genotoxic Stress Tolerance

**Figure 1:**
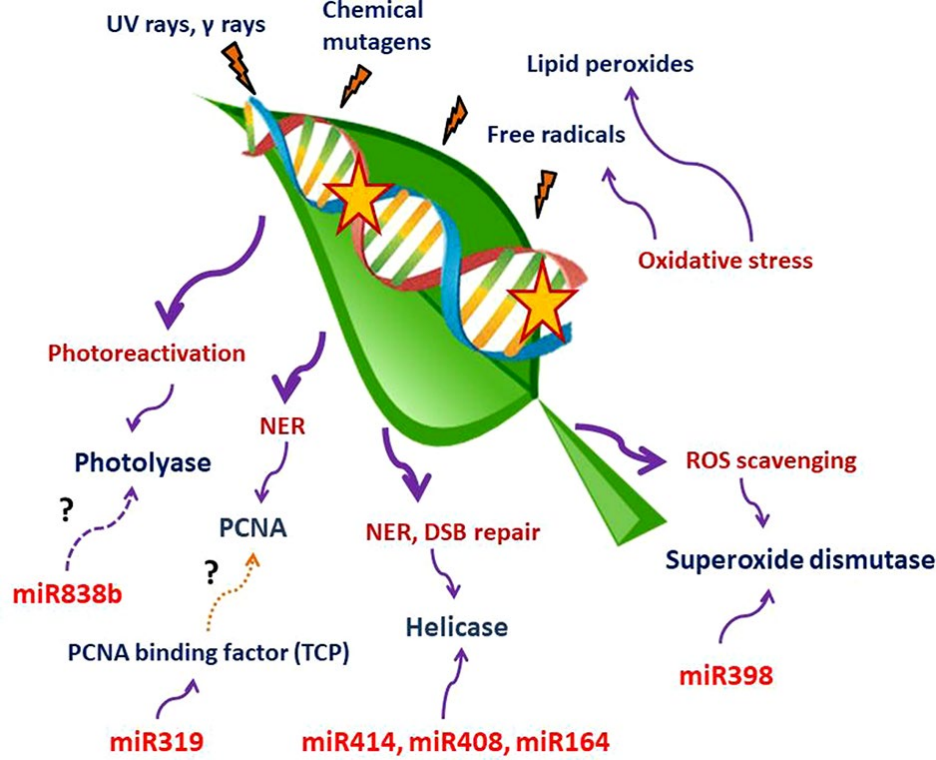
Schematic representation of miRNAs involvement in ROS scavenging and DNA repair mechanisms. UV and γ-rays, chemical mutagens, free radicals, and lipid peroxidation produced due to oxidative stress are among the most important DNA damage–causing agents. miR398, miR319, miR838b, miR414, miR408, and miR164 have been shown to target components involved in the abovementioned processes. The question mark indicates that more studies are required to establish the link with the adjacent pathways. There is no direct evidence of miR319 targeting TCPs that bind to the PCNA promoter (brown dotted line). Targeting of photolyase by miR838b through computational prediction needs experimental confirmation (magenta dashed line).

Till date, a handful of plant miRNAs with active role in combating genotoxic stresses have been identified in plants. Although the number is considerably low, we can divide these miRNAs into two categories. One set of miRNAs is involved in tolerating oxidative stress, while the other type of miRNAs may play an active part in DNA repair. A large set of enzymes acting as the key regulators of both ROS scavenging and DNA repair mechanism may be targeted by both these types of miRNAs. We have tabulated plant miRNAs found to target various enzymes involved in ROS scavenging and DDR network in **Table 1**. A schematic depiction of the involvement of miRNAs in these two processes is also shown in **Figure 1**.

**Table 1:** Involvement of miRNAs in targeting various enzymes involved in ROS scavenging as well as DDR network in plants.

Enzymes	miRNAs	Plant
Superoxide dismutase	miR398	*Arabidopsis* (Sunkar et al., 2006)
		Rice (Li et al., 2010)
		Wheat (Qiu et al., 2018), (Biselli et al., 2018)
		Grapevine (Leng et al., 2017)
		Barley (Xu et al., 2014b)
		Common bean (De la Rosa et al., 2019)
Photolyase	miR838b	*Brassica rapa* (Hajieghrari et al., 2017)
Helicases	miR414, miR408, miR164e	Rice (Macovei and Tuteja, 2013)
TCP gene	miR319	*Arabidopsis* (Koyama et al., 2017)

### miRNA Involvement in ROS Scavenging

SOD, a metal containing enzyme, is one of the most important enzymes for the removal of ROS produced by oxidative stress (Das and Roychoudhury, 2014). There are different types of SODs, including mitochondrial MnSOD, cytosolic and chloroplastic Cu/ZnSOD, and chloroplastic FeSOD (Van Camp et al., 1990; Bowler et al., 1994; Fukai and Ushio-Fukai, 2011). The complete regulatory mechanism of CSD1 and CSD2 is unknown, but recent studies have proved that miRNAs are having a role in this. Sunkar et al. (2006) have shown that the *Arabidopsis* ath-miR398 has a role in the regulation of CSD1 and CSD2. They found that downregulation of miR398 expression is important for the accumulation of CSD1/2 mRNA. While experimenting with different tissues, the same group of scientists have found that tissues like cauline leaves, stems, and roots (having high levels of miR398) showed lower expression of CSD1/2 mRNAs, whereas tissues like old rosette, leaves, and inflorescence (having low level of the same miRNAs) showed higher levels of CSD1/2 expression. Additionally, in rice, it was evidenced that miR159 targets Cu/ZnSOD (At5g18100-CSD3) (Li et al., 2010). Another group of scientists has shown that CSD is a potential targets of miR398 in wheat seedlings exposed to drought (Qiu et al., 2018). Cytosolic CSD1/2 was found to be targeted by miR398 in wheat in response to the fungal attack by *Fusarium graminearum* which causes *Fusarium* head blight disease (Biselli et al., 2018). Cytosolic CSD1 and chloroplastic CSD2 are potential targets of Vv-miR398 in grapevines (Leng et al., 2017). The Vv-miR398 family is highly conserved, and three loci, namely, miR398a (located on the chromosome no. 1), miR398b, and miR398c (located on the chromosome no. 6) encode the *MIR* gene in grapevine. In barley, hvu-miR398 was found to be negatively regulated by Mildew resistance locus a (Mla) and Mla resistance1 (rom1), and the overexpression of miR398 is responsible for the reduction in CSD1 (Xu et al., 2014b). In another experiment carried out in *Phaseolus vulgaris*, it has been shown that repression of miR398 leads to upregulation of CSD1 expression in case of water deficit (De la Rosa et al., 2019).

### miRNA Involvement in DNA Repair Mechanism

When considering the direct implication of miRNAs in plant DNA repair mechanisms, some helicases, important enzymes involved in both NER and DSB repairs, have been shown to be targeted by miR164, miR408, and miR414 in rice (Macovei and Tuteja, 2013). Several studies have addressed putative miRNA targeting mRNAs of genes involved in DDR by *in silico* analyses. For example, using psRNATarget server, a computational tool to predict miRNA targets, it was found that *Brassica rapa* miR838b putatively targets the photolyase mRNA (Hajieghrari et al., 2017). However, this prediction remains to be confirmed experimentally.

As an example of indirect involvement of miRNAs in DDR downstream processes, several studies established the contribution of miR319 to the regulation of TCP (teosinte-branched1/*Cincinnata*/proliferating cell factor) transcription factors, playing direct roles in leaf development (Danisman, 2016; Koyama et al., 2017; Bresso et al., 2018). It has been shown that two transcription factors, namely, PCF1 and PCF2, containing the non-canonical basic helix–loop–helix motif TCP domain, can regulate the transcription of PCNA (proliferating cell nuclear antigen) gene, an important component of the DDR network (Danisman, 2016; Nicolas and Cubas, 2016). Among the 24 members of *Arabidopsis* TCP family, miR319 targets the transcripts of *TCP2, TCP3, TCP4, TCP10*, and *TCP24* genes (Koyama et al., 2017). However, till date, there is no direct evidence showing that miR319 targeting TCPs can directly bind to the PCNA promoter.

## Challenges Related to MiRNA Applications in Combating Genotoxic Stresses in Plants

**Hurdle I**: Although many miRNAs have been found to be involved in the DDR network in human cells, very few miRNAs are found to be involved in DNA repair in plants. DNA repair processes are well characterized in mammalian systems; in contrast, very few studies are done in plants, and these are mainly limited to *Arabidopsis* and rice (Ueda and Nakamura, 2011; Macovei and Tuteja, 2013; Manova and Gruszka, 2015).

**Hurdle II**: There is absence of focused research on targeting genes involved in DNA repair even when considering miRNAs that are extensively studied in plants in relation to stress response; however, information about targets of miRNAs that are specifically involved in combating genotoxic stresses in plants is much more limited. Differently, many studies are being performed to find the targets of miRNAs in DDR in human diseases, including cancer studies (Hu and Gatti, 2011; He et al., 2016).

## How to Overcome the Challenges?

Identification of more plant miRNAs involved in DNA repair and ROS scavenging is one of the ways to overcome the challenge. In this regard, RNA sequencing of plants exposed to genotoxic stresses will lead to identification of new miRNAs specifically associated with genotoxic stresses. Once a substantial number of miRNAs are being identified in plants, their targets need to be validated. For target identification, bioinformatics approaches, along with experimental validation of the same by 5’ rapid amplification of cDNA ends (RACE), parallel analysis of RNA ends (PARE), degradome-seq, or genome-wide mapping of uncapped transcripts, can be of great help. References from miRNAs involved in DDR in human diseases can be taken, and extensive studies must be performed to find out the role of plant miRNAs in targeting the homolog proteins involved in DNA repair in plant species.

Several human diseases are found to be associated with miRNA-dependent regulation of DNA repair pathways. For example, RAD23 and CDK7 are two important enzymes in NER pathway. miR-494 was found to target RAD23 homolog B (Comegna et al., 2014), while CDK7 is targeted by miR-210 (Abdullah et al., 2016). Human miR-103a-2-5p and miR-585-5p target poly ADP-ribose polymerase (PARP), an important BER enzyme (Dluzen et al., 2017). Human miR-422a base pairs with MLH1 3′-untranslated region and suppresses the expression of the same which in turn downregulate MutLα, a key protein of the MMR (Mao et al., 2012). In colorectal cancer cells, miR-7 targets XRCC2, a core protein involved in HR (Xu et al., 2014a). In cancer cell lines, RAD51 and BRCA1/2 (breast/ovarian cancer susceptibility gene products) are key proteins responsible for catalyzing HR, and both the proteins are potential target of miR-103 and miR-107 (Huang et al., 2013). BRCA1/2 is also found to be targeted by miR-15/107/182 in breast cancer (Petrovic et al., 2017). MSH2, another essential MMR component, is downregulated by miR-21 in human (Valeri et al., 2010). Homologs of all the human RAD23, CDK7, PARP, MSH, MLH, XRCC2, RAD51, and BRCA1/2 are present in rice. However, there is no information available about potential miRNAs that target these mRNAs. While studying NHEJ repair in lung-cancer cell line, Yan et al. (2010) reported that miR-101 targets 3′- UTR of DNA-PKcs, a core component of NHEJ. In the same study, it has been proved that ATM, another key protein of HR-mediated repair, is a target of miR-101. Even tough plant homologues of ATM are reported in *Arabidopsis* and rice, no miRNA associated to their sequences was identified. Hence, it is noteworthy to mention that, even though it is well-established, several human genes involved in DNA repair are targeted by miRNAs, and some of their homologs are also reported in plants. No information is available about miRNAs targeting these genes in plants. With the advancement of genome annotation techniques and the availability of published and draft genomes, miRNAs can be searched firstly through bioinformatics approach. Once detected, these miRNAs can be experimentally verified for differential regulation of the target miRNAs associated with genotoxic stresses.

## Conclusion and Future Perspectives

DNA repair is a very important mechanism that allows plant cells to overcome genotoxic stresses and to maintain genome integrity. Impaired DNA repair mechanisms are the reason for plant slow growth and development, which in turn causes the reduction in crop production. In recent years, miRNAs have been identified as potentially novel and vital regulators of biological processes, including developmental processes and diseases. Considering their importance, it is essential to know more about miRNAs and their targets associated with DNA repair mechanism in plants. Once we find out the specific role of miRNAs and their targets in DNA repair and ROS scavenging, we could engineer them with genome-editing technologies like CRISPR-Cas, and hence aiming to combat a great number of genotoxic stresses.

## Author Contributions

MC wrote the manuscript. JB conceived the study and participated in its coordination.

## Conflict of Interest

The authors declare that the research was conducted in the absence of any commercial or financial relationships that could be construed as a potential conflict of interest.
